# The socio-demographic profile associated with perinatal depression during the COVID-19 era

**DOI:** 10.1186/s12889-023-15665-0

**Published:** 2023-04-28

**Authors:** Katina Kovacheva, María F. Rodríguez-Muñoz, Diego Gómez-Baya, Sara Domínguez-Salas, Emma Motrico

**Affiliations:** 1 Department of Personality, Assessment, and Psychological Treatment, Faculty of Psychology, Universidad Nacional de Educación a Distancia Madrid, Madrid, Spain; 2 Department of Social, Developmental and Educational Psychology, Universidad de Huelva, Huelva, Spain; 3 Department of Psychology, Universidad Loyola Andalucía, Dos Hermanas, Seville Spain

**Keywords:** Depression, Perinatal, COVID-19, Sociodemographic data

## Abstract

**Background:**

Coronavirus disease 2019 (COVID-19) has caused an increase in perinatal depression. The aim of this research was to identify which sociodemographic variables are related to the increase in perinatal depression due to the pandemic. In addition to estimating to what extent they predict perinatal depression, differentiating the prenatal and postnatal periods.

**Methods:**

The sample consisted of 3,356 subjects, 1,402 in the prenatal period and 1,954 in the postnatal period. The Edinburgh Postnatal Depression Scale was used to assess depressive symptomatology. A subset of 14 questions was included to collect demographic data. Items from the Spanish version of the Coronavirus Perinatal Experiences Survey were also included.

**Results:**

Experiencing the change of environment due to COVID-19 as negative and having a history of mental health predict perinatal depression, otherwise having higher education decreases the risk. In the prenatal stage having symptoms compatible with COVID-19 is a predictor of perinatal depression and having more than 3 years living together with the partner and being a housewife decreases the risk. In the postnatal stage being unemployed is a predictor of prenatal depression and being a first-time mother decreases the risk.

**Conclusions:**

This study highlights the relevance of sociodemographic status. It is essential to be aware of the risk factors of perinatal depression, to make adequate prevention, and to create health policies to alleviate the consequences of the pandemic.

**Supplementary Information:**

The online version contains supplementary material available at 10.1186/s12889-023-15665-0.

## Background

Perinatal depression (PPD) is a psychological disorder characterized by a non-psychotic depressive episode that occurs during pregnancy or in the first year after childbirth [1]. Non-detection or inadequate treatment leads to adverse effects on the physical and psychological health of the mother, the baby, difficulties in the maternal-filial bond, and family life [2], and for the healthcare system [3]. It is associated with adverse perinatal outcomes, including increased risk of poor adherence to medical care, poor nutrition, and smoking and substance abuse. It also impairs the mother's ability to be responsive to her baby's needs, affecting maternal-infant bonding and the baby's adaptive development [1].

In the scientific literature, several sociodemographic variables stand out as risk factors for PPD [4]. Age, primiparity, unemployment, not having a partner [5] poverty or domestic [6], and educational level [7, 8] have resulted in relevant. Existing reviews also agree that previous history of depression or anxiety and poor partner support are strong predictors of PPD [4].

Environmental conditions, such as extreme stress or emergencies are a potential predictor of the development of PPD [9, 10]. During the Coronavirus disease 2019 (COVID-19) pandemic, the number of publications on this topic has grown exponentially, although not always clear. However, all indicate that the prevalence of PPD has doubled [11]. Went from a global prevalence of 10–13% in the previous years [9] to 22–31% during the pandemic [12, 13]. However, these studies have some limitations, such as small and geographically unrepresentative samples, administration of non-validated assessment instruments, and non-differentiation of the prenatal and postnatal period [14].

The measures imposed to stop the spread of the pandemic, due to COVID-19, have caused multiple socioeconomic problems, which are considered a risk to mental health [9]. It has been observed that material and socioeconomic conditions during the pandemic had a greater impact on women's mental health [15], moreover childbearing years are the period with the highest peak prevalence of major depressive disorder [16]. Among the variables explaining the increase in PPD are mentioned, fear of infection [17], high levels of COVID-19-related health concerns, and high levels of COVID-19-related grief [18]. It was also observed increase of PPD prevalence, as the number of new confirmed infections, the number of supposed infections, and the number of new deaths per day raised [19]. These variables, age, socioeconomic status, and education level [19], are consistent with existing pre-pandemic research [8].

Research regarding the impact of the pandemic on perinatal mental health is still scarce, key questions remain to be answered [20], making it necessary to develop new studies. This research thus aims to contribute to a more precise understanding of the influence of the pandemic on PPD and to generate recommendations for the creation of specific policies to protect perinatal mental health. For this purpose, the objectives of this study were: 1) to identify sociodemographic characteristics related to PPD during the COVID-19 era; 2) to estimate the extent to which observed risk factors predict the development of PPD; 3) to assess differences between the prenatal and postnatal period in these risk factors for PPD.

## Method

### Design

This research is part of an international study on the impact of the COVID-19 pandemic on perinatal mental health (Riseup-PPD-COVID-19), with a published study protocol (trial registry: ClinicalTrials.gov; Identifier: NCT04595123), where the method is described in detail. Data collected in Spain were used for this work. Specifically, a cross-sectional study was used to examine risk factors for PPD among pregnant women and mothers of infants younger than 6 months.

STROBE Statement for reporting cross-sectional studies was followed [21] (see checklist in Supplementary material S1).

### Participants and procedure

The sample included 1,402 pregnant women and 1,954 postpartum women residing in Spain, recruited between June 16 and December 04, 2020.

Inclusion criteria were: i) being pregnant or a biological mother of a child aged 6 months or younger; ii) were 18 years of age or older; iii) residing in Spain at the time of participation in the study; and iv) accepting informed consent.

### Procedure

The present study received approval from the Ethics Committee (Ethics Protocol: 1257-N-20). All data were completely anonymized, according to the Helsinki Declaration of Research with Human Beings.

Participants were recruited through social media, organizational networks, policymakers, local organizations, and other interested parties.

Before completing the questionnaire from the project website link (https://momsduringcovid.org), participants were informed about the objectives of the study, the content of the questions, the potential risks and benefits, and the ethical aspects of the study. Data collection took approximately 20 min to complete.

### Instruments

The Edinburgh Postnatal Depression Scale (EPDS) [22], an instrument designed to detect postpartum depression [23], was used to assess depressive symptomatology. It consists of 10 items with four response options, whose scores range from 0 to 3. It measures symptoms of sadness, anxiety, and thoughts about death. Scores range from 0 to 30, with higher scores indicating greater severity. The EPDS is the most internationally recommended and used scale, but it does not have a specified cut-off point [24]. In this study we used the Spanish validation [25] with a cut-off point of 13 or more to identify clinically significant symptoms, assuming a lower sensitivity, but higher specificity [24]. The reliability obtained from the EPDS was 0.89 during pregnancy and 0.88 postpartum.

To collect demographic data, we included a subset of 14 questions on women's date and country of birth; state/city of residence; educational level; the number of previous pregnancies; the number of biological children (including those stillborn); the number of people living in the household (adults and children); marital status; cohabitation with a partner; household characteristics (indoor and outdoor spaces in square meters); changes in living environment since the onset of the pandemic; and mental history.

The Coronavirus Perinatal Experiences—Impact Survey (COPE-IS) is a global measure created to assess the experiences of pregnant and postpartum women during the COVID-19 pandemic [26]. COPE-IS, initially written in English, has been adapted into other languages (for example, German, Portuguese, and Spanish). Thus, researchers from each country involved in the study performed the translation and cultural adaptation of the questionnaires from English into the official language of their country, following several methodological steps defined a priori, as detailed in the study protocol [14]. Psychometric properties for the measure have yet to be established. Specifically in this study, we selected four item from the scale dealing with (1) diagnosis of COVID-19; (2) any symptoms compatible with coronavirus disease; (3) contact with someone diagnosed with COVID-19; (4) any death of family or friends due to COVID-19; and two items on current employment status.

### Statistical analysis

Statistical analysis was performed using IBM SPSS Statistics for Windows (version 24), setting the confidence level at 95% and the significance levels at 1% and 5% (*p* < 0.01 and *p* < 0.05). Two groups were considered in the analysis: pregnant women and postpartum women. After manually verifying the 4,316 respondents, 960 records were eliminated because they indicated an erroneous duration of pregnancy (more than 42 weeks; *N* = 324) or their children were more than 6 months old (*N* = 636). PPD (criterion variable) was measured dichotomously with EPDS > 13. Records with no missing values were used for all analyses. Differences between the presence or absence of depression were analyzed to the categorical variables in each of the groups, contingency tables were made and the Pearson Chi-Square statistic was applied. For the variables with mean scores obtained in the scales, mean comparisons were made and Student's t-test for independent samples was used. The Cramer's V effect size index or Cohen's d were also presented following the interpretation scale of 0–0.19, insignificant; 0.20–0.49, small; 0.50–0.79, medium; more than 0.80, high [27].

A stepwise binary logistic regression analysis was carried out with each of the groups to find out the sociodemographic variables and COVID-19-related variables that predict depression in pregnancy and postpartum. This analysis allows the prediction of the dichotomous dependent variable (with depression/without depression) as a function of relevant or influential predictor variables (categorical or quantitative).

## Results

### Characteristics of the participants

The total sample participants were 3,356 subjects, 1,402 pregnant women, and 1,954 postpartum women. The sample was drawn from the 17 Spanish autonomous communities and the two autonomous cities (Ceuta and Melilla). Most of them lived in Andalusia (18%), Madrid (17.20%), Catalonia (13.60%), and Valencia (7.60%). Table 1 shows the sociodemographic characteristics. The mean age of the full sample was 33.67 years (± 4.25). More than half had a university education (71.70%) and were first-time mothers (62.10%). Most lived with a partner (80.30%), were employed (89.20%), and reported no previous history of depression (79.30%).

**Table 1:** Main characteristics of the participants

Sociodemographic variables	All participants (*N* = 3,356) *N (%)*	Pregnant women (*n* = 1,402) *n (%) o Mean (SD)*	Postpartum women (*n* = 1,954) *n (%) o Mean (SD)*
Age	33.67 (4.25)	33.43 (4.18)*	33.84 (4.29)*
Education Level			
Basic education	160 (4.80%)	62 (4.50%)	98 (5.10%)
Intermediate studies	780 (23.50%)	300 (21.60%)	480 (24.90%)
Higher education	2,377 (71.70%)	1,025 (73.90%)	1,352 (70.10%)
Primiparous			
*Yes*	2,083 (62.10%)	832 (59.30%)*	1,251 (64%)*
*No*	1,273 (37.90%)	570 (40.70%)*	703 (36%)*
Cohabitation with a partner			
*Yes*	2,695 (81.40%)	1,143 (81.55%)	1,552 (80.70%)
*No*	615 (18.60%)	245 (17.45%)	370 (19.30%)
Mental Health History			
*Yes*	695 (20.70%)	312 (22.30%)	383 (19.60%)
*No*	2,661 (79.30%)	1,090 (77.70%)	1,571 (80.40%)
Employment			
*Employed*	363 (10.80%)	154 (11%)	209 (10.70%)
*Unemployed*	2,993 (89.20%)	1,248 (89%)	1,745 (89.30%)

^*^ *p*-value < 0.05

### Descriptive and comparative analysis

Table 2 shows the comparison of sociodemographic characteristics in the prenatal period between women with clinically significant symptoms of depression and those without. The results showed significant differences concerning mental health history, unemployment status, having a full-time job, educational level, age, time living with the partner, being a first-time mother, having symptoms compatible with COVID-19, change of environment experienced since the beginning of the pandemic, number of people living together in the household, and number of children in the household. Specifically, among women with suspected depression, the percentage of those who have a history of mental health is higher (36% vs. 16.90%), are unemployed (15.01% vs. 9.41%), do not have a full-time job (57.40% vs. 47.10%), have only primary or secondary education (7.20% vs. 3.40%), are under 26 years of age (7.10% vs. 3.30%) have been living with their partner for less than 3 years (26.18% vs. 17.70%), are not first-time mothers (44.90% vs. 39%), have symptoms compatible with the COVID-19 virus (20.40% vs. 13.40%), have experienced the change of environment since the beginning of the pandemic as very negative (24.30% vs. 8.70%), live with a greater number of people in the household (2.66 vs. 2.50) and have a greater number of children (0.56 vs. 0.46).

**Table 2:** Descriptive and comparative analysis between pregnant women with EPDS and non EPDS

	**EPDS < 13** **(** ***n*** ** = 1,010)** **n (%) or Mean (SD)**	**EPDS ≥ 13** **(** ***n*** ** = 392)** **n (%) or Mean (SD)**	***p***	***Effect size***
Mental Health History				
*Yes*	171 (16.90%)	141 (36%)	< 0.001	0.205
*No*	839 (83.10%)	251 (64%)		
Age range				
*18—26*	32 (3.30%)	27 (7.10%)	0.007	0.086
*27—36*	710 (72.50%)	269 (71%)		
*37—49*	237 (24.20%)	83 (21.90%)		
Education Level				
*Basic education*	34 (3.40%)	28 (7.20%)	< 0.001	0.119
*Intermediate studies*	197 (19.70%)	103 (26.60%)		
*Higher education*	769 (76.90%)	256 (66.10%)		
Partner				
*Yes*	963 (96.10%)	369 (95.30%)	0.523	0.017
*No*	39 (3.90%)	18 (4.70%)		
Cohabitation with a partner				
*Yes*	823 (82.20%)	320 (82.70%)	0.837	0.006
*No*	178 (17.80%)	67 (17.30%)		
Time living with a partner				
< *3 years*	143 (17.70%)	83 (26.20%)	0.006	0.096
*3—6 years*	298 (36.80%)	108 (34.10%)		
> *6 years*	368 (45.50%)	126 (39.70%)		
Primiparous				
*Yes*	616 (61%)	216 (55.10%)	0.044	0.054
*No*	394 (39%)	176 (44.90%)		
Unemployment status				
*Yes*	95 (9.40%)	59 (15.10%)	0.002	0.081
*No*	915 (90.60%)	333 (84.90%)		
Full Time Work				
*Yes*	534 (52.90%)	167 (42.60%)	< 0.001	0.092
*No*	476 (47.10%)	225 (57.40%)		
Homemaker				
*Yes*	59 (5.80%)	23 (5.90%)	0.985	< 0.001
*No*	951 (94.20%)	369 (94.10%)		
Looking for work				
*Yes*	28 (2.80%)	8 (2%)	0.437	0.021
*No*	982 (97.20%)	384 (98%)		
Paid Maternity Leave				
*Yes*	138 (13.70%)	58 (14.80%)	0.583	0.015
*No*	872 (86.30%)	334 (85.20%)		
Number of children	1.27 (0.66)	1,29 (0.61)	0.652	0.035
Number of persons living in household	2.5 (0.86)	2,66 (1)	0.003	0.176
Number of children living in household	0.46 (0.75)	0,56 (0.80)	0.027	0.132
Number of adults living in the home	1.87 (0.63)	1,95 (0.68)	0.069	0.112
Meters outside house space	85.48 (484.48)	137.12 (991.46)	0.191	0.079
House interior meters				
< *80 m2*	310 (32%)	122 (32.90%)	0.948	0.009
*81 m2—110 m2*	424 (43.71%)	161 (43.40%)		
> *111 m2*	235 (24.29%)	88 (23.70%)		
Confirmed COVID-19 diagnosis				
*Yes*	68 (6.70%)	28 (7.10%)	0.785	0.007
*No*	942 (93.30%)	364 (92.90%)		
Symptoms COVID-19 diagnosis				
*Yes*	135 (13.40%)	80 (20.40%)	0.001	0.088
*No*	875 (86.60%)	312 (79.60%)		
Contact with someone who has been diagnosed with COVID-19				
*Yes*	266 (26.30%)	120 (30.60%)	0.108	0.043
*No*	744 (73.70%)	272 (69.40%)		
Death of a close person due to COVID-19				
*Yes*	109 (10.80%)	55 (14%)	0.090	0.045
*No*	901 (89.20%)	337 (86%)		
Change in environment since start of pandemic				
*Very positive*	31 (3.20%)	3 (0.80%)	< 0.001	0.253
*Somewhat positive*	125 (12.70%)	31 (8.10%)		
*No impact*	205 (20.80%)	32 (8.40%)		
*Somewhat negative*	537 (54.60%)	224 (58.50%)		
*Very negative*	86 (8.70%)	93 (24.30%)		

Table 3 shows the comparison in the postnatal period between women who have significant symptoms of depression and those who do not. The results showed significant differences concerning mental health history, living with a partner, being unemployed, job search, having a full-time job, being on paid maternity leave, being a homemaker, level of education, losing a close relative who died due to COVID-19, and change in the environment experienced since the beginning of the pandemic. Specifically, among women with suspected depression, the percentage of those with a history of mental health is higher (29.40% vs. 15.60%), are not living with their partner (22.45% vs. 17.87%), are unemployed (15.70% vs. 8.55%), are looking for a job (6.80% vs 3.90%), do not have a full-time job (67.90% vs 61.20%), do not have paid maternity leave (47.27% vs 37.03%), are housewives (12.10% vs 7.67%), have only primary or secondary education (9.42% vs 3.32%), have lost a close person due to COVID-19 (13.20% vs 9.28%), and have not experienced a change in environment since the beginning of the pandemic (29.40% vs 9.80%).

**Table 3:** Descriptive and comparative analysis between postpartum women with PPD and those without PPD

	**EPDS < 13** **(** ***n*** ** = 1,369)** **n (%) or Mean (SD)**	**EPDS ≥ 13** **(** ***n*** ** = 585)** **n (%) or Mean (SD)**	***p***	***Effect size***
Mental Health History				
*Yes*	211 (15.40%)	172 (29.40%)	< 0.001	0.161
*No*	1.158 (84.60%)	413 (70.60%)		
Age range				
*18—26*	46 (3.50%)	32 (5.70%)	0.853	0.054
*27—36*	938 (71%)	380 (67.50%)		
*37—49*	338 (25.60%)	151 (26.80%)		
**Education Level**				
*Basic education*	44 (3.32%)	54 (9.42%)	< 0.001	0.158
*Intermediate studies*	306 (22.70%)	174 (30%)		
*Higher education*	1,000 (74.10%)	352 (60.58%)		
Partner				
*Yes*	1,274 (94.37%)	544 (93.79%)	0.619	0.011
*No*	76 (5.63%)	36 (6.21%)		
Cohabitation with a partner				
*Yes*	1,103 (82.13%)	449 (77.55%)	0.019	0.053
*No*	240 (17.87%)	130 (22.45%)		
Time living with a partner				
< *3 years*	173 (15.90%)	60 (13.69%)	0.208	0.033
*3—6 years*	359 (3.90%)	141 (32%)		
> *6 years*	558 (51.20%)	239 (54.31%)		
**Primiparous**				
*Yes*	891 (65.10%)	360 (61.50%)	0.135	0.034
*No*	478 (34.90%)	225 (38.50%)		
Unemployment status				
*Yes*	117 (8.55%)	92 (15.70%)	< 0.001	0.106
*No*	1.252 (91.45%)	493 (84.30%)		
Full Time Work				
*Yes*	531 (38.79%)	188 (32.10%)	0.005	0.071
*No*	838 (61.21%)	397 (67.90%)		
Homemaker				
*Yes*	105 (7.67%)	71 (12.10%)	0.002	0.040
*No*	1,264 (92.33%)	514 (87.90%)		
Looking for work				
*Yes*	54 (3.94%)	40 (6.80%)	0.006	0.063
*No*	1,315 (96.06%)	545 (93.20%)		
Paid Maternity Leave				
*Yes*	862 (62.96%)	309 (52.73%)	< 0.001	0.062
*No*	507 (37.03%)	276 (47.27%)		
Number of children	1.49 (0.77)	1.45 (0.74)	0.233	0.059
Number of persons living in household	3.51 (0.88)	3.56 (0.94)	0.319	0.050
Number of children living in household	1.42 (0.87)	1.44 (0.76)	0.488	0.034
Number of adults living in the home	2.06 (0.54)	2.08 (0.73)	0.620	0.028
Meters outside house space	84.85 (438.01)	119.41 (699.20)	0.277	0.065
House interior meters				
< *80 m2*	395 (30.10%)	173 (30.84%)	0.567	0.015
*81 m2—110 m2*	559 (45.70%)	260 (46.34%)		
> *111 m2*	318 (24.20%)	128 (22.82%)		
Confirmed COVID-19 diagnosis				
*Yes*	84 (6.10%)	29 (5%)	0.307	0.023
*No*	12,85 (93.90%)	556 (95%)		
Symptoms COVID-19 diagnosis				
*Yes*	174 (12.71%)	80 (13.70%)	0.561	0.013
*No*	1,195 (87.29%)	505 (86.30%)		
Contact with someone who has been diagnosed with COVID-19				
*Yes*	345 (25.20%)	142 (24.30%)	0.664	0.010
*No*	1,024 (74.80%)	443 (75.70%)		
Death of a close person due to COVID-19				
*Yes*	127 (9.28%)	77 (13.20%)	0.010	0.058
*No*	1,242 (90.72%)	508 (86.80%)		
Change in environment since start of pandemic				
*Very positive*	48 (3.60%)	9 (1.60%)	< 0.001	0.270
*Somewhat positive*	172 (13%)	53 (9.30%)		
*No impact*	227 (17.10%)	39 (6.90%)		
*Somewhat negative*	747 (56.40%)	300 (52.80%)		
*Very negative*	130 (9.81%)	167 (29.40%)		

To determine which variables predict PPD, a stepwise binary logistic regression analysis was performed. As a dependent variable, the EPDS scale dichotomized according to the cut-off point (yes depression / no depression) was used, and as independent variables were included: in the first model, mental history (yes/no); in the second model, sociodemographic data on educational level (basic/medium/higher), age (18–26/27–36/ > 37), being a first-time mother (yes/no), time living with the partner (< 3 years/ 3–6 years/ > 3 years), unemployment status (yes/no), full-time job (yes/no), paid maternity leave (yes/no), housewife (yes/no), job search (yes/no) were added; and in a third model we also incorporated data related to COVID-19 such as having presented symptoms compatible with COVID-19 (yes/no), losing a close relative due to COVID-19 (yes/no), changes in the environment experienced due to COVID-19 (very positive/somewhat positive/no impact/somewhat negative/very negative) and the number of people and children living together in the household (n).

For the selection of the dependent variables, we selected those we presumed to be relevant based on the scientific literature and thus those observed as influential in the previous comparative analysis.

All models were significant (*p* < 0.01) so the models presented a good fit to the data. Table 4 indicates what proportion of the variability of the depression variable could be controlled using each model in pregnant women and postpartum women.

**Table 4:** R2 value of the binary logistic regression models in the sample of pregnant and postpartum women

**Model**		**Pregnant women**	**Postpartum women**
Model 1. Mental Health History	Cox and Snell R-squared	0.04	0.02
	Nagelkerke R-squared	0.06	0.04
Model 2. Sociodemographic factors	Cox and Snell R-squared	0.07	0.05
	Nagelkerke R-squared	0.10	0.07
Model 3. COVID-19 factors	Cox and Snell R-squared	0.13	0.11
	Nagelkerke R-squared	0.18	0.16

As can be seen in Table 5 the results found show that experiencing the change of environment due to COVID-19 is very negative (OR = 12.96) or somewhat negative (OR = 4.57), having a mental history (OR = 2.49), having symptoms compatible with COVID-19 (OR = 1.77) are predictors of depression during pregnancy. Living with the partner for 3 to 6 years (OR = 0.67) or more than 6 years (OR = 0.56), having higher education (OR = 0.48), and being a housewife (OR = 0.42) decrease the risk of depression. It should also be noted that in Model 2, in which factors related to COVID-19 were not included, being a first-time mother was a protective factor (OR = 0.67), and being a housewife was not relevant.

**Table 5:** Regression analysis of predictors of depression among pregnant women

Predictors	Model 1		Model 2		Model 3	
	**Mental Health History**		**Sociodemographic factors**		**COVID-19 factors**	
	**OR [95% IC]**	***p***	**OR [95% IC]**	***p***	**OR [95% IC]**	***p***
Mental Health History	2.75 [2.11–3.58]	** < 0.001**	2.63 [1.92—3.59]	** < 0.001**	2.49 [1.80—3.45]	** < 0.001**
Age 18—26 years				0.691		0.694
Age 27—36 years			0.83 [0.39—1.76]	0.640	0.76 [0.34—1.66]	0.497
Age > 37 years			0.74 [0.33—1.65]	0.468	0.70 [0.30—1.61]	0.405
*Basic education*				**0.008**		**0.006**
*Intermediate studies*			0.93 [0.45—1.90]	0.849	0.81 [0.38—1.70]	0.580
*Higher education*			0.56 [0.28—1.11]	0.102	0.48 [0.23—0.98]	**0.044**
Cohabitation with a partner < 3 years				**0.009**		**0.019**
Cohabitation with a partner 3—6 years			0.62 [0.42—0.91]	**0.015**	0.67 [0.45—0.99]	**0.047**
Cohabitation with a partner > 6 years			055 [0.37—0.81]	**0.003**	0.56 [0.37—0.84]	**0.005**
Unemployment status			1.54 [0.96—2.46]	0.068	1.58 [0.97—2.57]	0.065
Full time work			0.85 [0.62—1.16]	0.314	0.84 [0.61—1.16]	0.291
Homemaker			0.52 [0.27—1.02]	0.060	0.42 [0.21—0.86]	**0.019**
Looking for work			0.66 [0.25—1.71]	0.395	0.63 [0.24—1.66]	0.360
Paid Maternity Leave			1.00 [0.67—1.50]	0.988	1.04 [0.68—1.58]	0.831
Primiparous			0.67 [0.49—0.92]	**0.014**	0.91 [0.58—1.44]	0.715
Number of persons living in household					0.99 [0.75—1.31]	0.974
Number of children living in household					1.31 [0.87—1.99]	0.191
Symptoms COVID-19 diagnosis					1.77 [1.20—2.62]	**0.004**
Death of a close person due to COVID-19					1.24 [0.81—1.89]	0.307
Change in environment since start of pandemic Very positive						** < 0.001**
Somewhat Positive					3.34 [0.72—15.40]	0.121
No Impact					2.00 [0.43—9.20]	0.370
Somewhat Negative					4.57 [1.04—20.05]	**0.044**
Very negative					12.96 [2.86—58.60]	**0.001**

As can be seen in Table 6, the results show that experiencing the change of environment as very negative due to COVID-19 (OR = 6.64), having a mental history (OR = 2.03), and being unemployed (OR = 1.58) are predictors of postpartum depression. Being a first-time mother (OR = 0.66), and having middle (OR = 0.44) or higher education (OR = 0.32) decreased the risk of depression. In Model 2, which does not include factors related to COVID-19, being a first-time mother was not a relevant factor.

**Table 6:** Regression analysis of predictors of depression among the sample of postpartum women

Predictors	Model 1		Model 2		Model 3	
	**Mental Health History**		**Sociodemographic factors**		**COVID-19 factors**	
	**OR [95% IC]**	***p***	**OR [95% IC]**	***p***	**OR [95% IC]**	***p***
Mental Health History	2.28 [1.81—2.87]	** < 0.001**	2.08 [1.58—2.73]	** < 0.001**	2.03 [1.53—2.70]	** < 0.001**
Age 18—26 years				0.488		0.253
Age 27—36 years			0.84 [0.43—1.64]	0.617	0.78 [0.38—1.61]	0.511
Age > 37 years			0.98 [0.48—1.97]	0.956	0.97 [0.46—2.07]	0.952
*Basic education*				** < 0.001**		** < 0.001**
*Intermediate studies*			0.49 [0.27—0.88]	**0.018**	0.44 [0.24—0.83]	**0.011**
*Higher education*			0.35 [0.20—0.63]	** < 0.001**	0.32 [0.17—0.59]	** < 0.001**
Cohabitation with a partner < 3 years				0.407		0.354
Cohabitation with a partner 3—6 years			1.22 [0.84—1.77]	0.296	1.20 [0.81—1.77]	0.359
Cohabitation with a partner > 6 years			1.28 [0.89—1.84]	0.181	1.32 [0.90—1.93]	0.153
Unemployment status			1.71 [1.14—2.56]	**0.009**	1.58 [1.04—2.40]	**0.032**
Full time work			0.85 [0.65—1.10]	0.228	0.85 [0.65—1.11]	0.245
Homemaker			0.95 [0.62—1.45]	0.828	0.94 [0.60—1.46]	0.789
Looking for work			0.86 [0.48—1.54]	0.622	0.86 [0.47—1.59]	0.649
Paid Maternity Leave			0.83 [0.64—1.07]	0.157	0.80 [0.61—1.04]	0.097
Primiparous			0.88 [0.68—1.14]	0.342	0.66 [0.46—0.94]	**0.024**
Number of persons living in household					0.78 [0.59—1.03]	0.081
Number of children living in household					1.02 [0.76—1.37]	0.872
Symptoms COVID-19 diagnosis					0.91 [0.64—1.31]	0.634
Death of a close person due to COVID-19					1.45 [0.99—2.12]	0.053
Change in environment since start of pandemic Very positive						** < 0.001**
Somewhat Positive					1.91 [0.78—4.68]	0.155
No Impact					0.90 [0.35—2.25]	0.824
Somewhat Negative					2.21 [0.95—5.11]	0.064
Very negative					6.64 [2.78–15.86]	** < 0.001**

## Discussion

Despite the widely studied relationship in the literature between depression and certain sociodemographic variables, to our knowledge, there are hardly any studies on these variables in the situation caused by the COVID-19 pandemic. The limited evidence suggests adverse effects on mental health due to this pandemic period [28]. The impact of confinement on mental health is related to gender, age, and socioeconomic conditions, with a higher proportion of anxiety and depressive symptoms among women aged 18–35 years and with worsening economic conditions [15]. Therefore, it is important to examine the sociodemographic data in this study population.

Given the adverse effects of PPD, there is an urgent need to assess risk and protective factors during the pandemic, which was the aim of this study, to minimize the consequences on women in the perinatal period [29] and reduce health inequalities [15].

Regarding the predictor variables of PPD, in our results, mental health antecedents have an important weight throughout the perinatal stage. Thus, coinciding with previous studies and highlighting the higher risk of PPD in mothers with a previous history of depression [7]. However, the most relevant variable, also in the entire perinatal stage, and in turn the most innovative data of this study, is the weight of the subjective evaluation of the change of environment since the beginning of the pandemic. In the case of pregnant women, those who perceive the change of environment as very negative have 12.96 times more risk of developing depression and at the time of postpartum have 6.64 times more risk. The negative perception of environmental change could be generating life stress, which is a potential predictor of perinatal depression in both bivariate and multivariate analyses [10]. These data evidence the importance of changing the way we assess the environment as a key component in clinical practice.

In addition to those already mentioned, another predictor variable for PPD during pregnancy is having symptoms compatible with COVID-19. These data are consistent with those of Liu [18] who reported that high levels of COVID-related health concerns pose a risk factor. In contrast, the variables that decrease the risk of having PPD during pregnancy are being a homemaker and having been cohabiting with the partner for more than three years, the latter also observed by Sahin & Seven [30]. Regarding the variable being a housewife, it has generally been associated as a risk factor [31], due to its relationship with having a smaller support network, some isolation [32], and lower economic resources [31]. However, considering the importance of the variable perception of environmental change due to COVID-19, it is possible that the measures imposed to stop the pandemic may not have meant so much change and uncertainty for housewives. The regression analysis model does not include the variables about COVID-19, being a housewife is not a relevant variable.

In postpartum another predictor variable of PPD is unemployment status, also in line with previous studies [4, 6, 7, 19]. This may be because higher economic status facilitates access to health services, childcare, and information [33]. Conversely, being a first-time mother decreases the risk of PPD. It is a variable generally considered a risk factor for PPD [4], but with some contradictory results, as some studies associate multiparity with increased risk of depression, which may be due to a higher burden of care and added psychosocial stress [34]. In our results, this variable becomes relevant when we include variables related to COVID-19 in the regression analysis, so they seem to modulate it.

As far as the educational level is concerned, it supposes a protective factor throughout the perinatal stage, in consensus among researchers, in which lower educational level has always been associated with PPD [6, 7, 19].

Another result to highlight from the analysis of the differences between depressed and non-depressed pregnant women is that the mean number of people and the mean number of children cohabiting during the pandemic is higher among pregnant women with depression. This fact was also observed by Gonzalez-Mesa et al. [35], who found that women cohabiting with more people in the same household are at higher risk of antenatal depression. During the pandemic, it has been observed that there is a relationship between having a worse perception about housing with the size of square meters per person [15]. Regarding the analysis of differences between postpartum women with depression and those without depression in our sample, a higher percentage of women with depression were housewives, without access to paid maternity leave, seeking employment, and not living with a partner. These factors are also in line with previous studies [4, 8, 19]. The importance of the economic situation stands out, especially in the postpartum period when the woman returns to the need to work. In this sample, the most interesting finding is that among the mothers with depression they have experienced to a greater extent the death of a close relative due to COVID-19, data in line with those of Liu et al. [18], who related high levels of bereavement as risk factors.

Therefore, from the results found in this sample, in relation to the pandemic, we can highlight among the variables associated with PPD during pregnancy the concern of having COVID-19, the number of people and children living together at home, and the perception of the change of environment due to COVID-19 as very negative. On the other hand, during the postnatal stage, having suffered the loss of a close member due to COVID-19 and the perception of the change of environment due to COVID-19 as very negative were associated. The explanation for these findings could be that during pregnancy the woman is focused on her health and that of her baby, whereas during the postpartum period the need for the presence of support figures is more important.

Considering that prenatal depression is the main risk factor for postpartum depression [8], it is essential to carry out preventive work from pregnancy onwards and to consider the different risk factors at each stage. Therefore, we hope that the data provided in this study will help perinatal healthcare personnel to develop preventive measures or, if necessary, provide early care. Especially when there is evidence indicating the possibility of preventing depressive episodes with interventions administered by both trained and untrained professionals [36].

The results should be interpreted taking into consideration certain limitations such as the fact that the sample is not a random probability sample, since unintentional sampling was used. In addition, the possibility of internet access or educational level may have limited the participation of some groups. Adding that the survey could add people who are more interested in this topic or who are more affected by psychological factors.

On the other hand, the strength of the study is the size of the sample and its relevance given the current situation. Knowledge of the risk and protective factors, differentiating the perinatal period, before and after childbirth, is fundamental for adequate prevention of depression.

It is important to continue to conduct further studies on the impact of the pandemic on perinatal health and to take the results into account for clinical practice in the coming years and any future emergency crisis. As has been observed in previous epidemics, the impact generated can last for a long period and have long-term consequences on mental health [15]. In addition, the experience of two or more stressful events in the previous year of pregnancy has been related to PPD [37]. From there, it is important for health professionals to be aware of the increased risk of PPD and to use referral pathways if necessary [38]. Just as an intersectoral approach has been chosen to stop the pandemic, all policies should continue in this line of work to mitigate the consequences. The strategic lines developed in response to the health crisis should emphasize psychological care during COVID-19 and in subsequent phases [39].

## Conclusions

To conclude, the COVID-19 pandemic has had a profoundly devastating effect on perinatal mental health. This study highlights the relevance of the sociodemographic situation, demonstrating that if health equity is to be increased, it is necessary to develop programs aimed at people who are disadvantaged in terms of the full development of their health potential.

## Supplementary Information

**Additional file 1. **STROBE Statement—checklist of items.

## Data Availability

The datasets generated and/or analyzed during the current study are not publicly available due to legal reasons but are available from the corresponding author upon reasonable request.

## Abbreviations

PPD: Perinatal depression
COVID-19: Coronavirus 2019
EPDS: The Edinburgh Postnatal Depression Scale
COPE-IS: Coronavirus Perinatal Experiences Survey
