# Supplementary material for: The socio-demographic profile associated with perinatal depression during the COVID-19 era
Source: BMC Public Health. 2023 Apr 28;23:786. doi: 10.1186/s12889-023-15665-0 (PMC10141819; doi:10.1186/s12889-023-15665-0)
Supplement: Supplementary file 1 — Additional file 1. STROBE Statement—checklist of items. [file 12889_2023_15665_MOESM1_ESM.docx]

STROBE Statement—checklist of items

|  | Item | Recommendation |  |
| --- | --- | --- | --- |
| **Title and abstract** | 1 | (*a*) Indicate the study’s design with a commonly used term in the title or the abstract | In the abstract, section objective |
|  |  | (b) Provide in the abstract an informative and balanced summary of what was done and what was found |  |
| **Introduction** |  |  |  |
| Background / rationale | 2 | Explain the scientific background and rationale for the investigation being reported | In the introduction |
| Objectives | 3 | State specific objectives, including any prespecified hypotheses | At the end of the introduction |
| **Methods** |  |  |  |
| Study design | 4 | Present key elements of study design early in the paper | In the method, design and participants section |
| Setting | 5 | Describe the setting, locations, and relevant dates, including periods of recruitment, exposure, follow-up, and data collection | In the method, participants and procedure section |
| Participants | 6 | Cross-sectional study—Give the eligibility criteria, and the sources and methods of selection of participants | In the method, participants and procedure section |
| Variables | 7 | Clearly define all outcomes, exposures, predictors, potential confounders, and effect modifiers. Give diagnostic criteria, if applicable | In the method, statistical analysis section |
| Data sources / measurement | 8 | For each variable of interest, give sources of data and details of methods of assessment (measurement). Describe comparability of assessment methods if there is more than one group | In the method, instruments and statistical analysis section |
| Bias | 9 | Describe any efforts to address potential sources of bias |  |
| Study size | 10 | Explain how the study size was arrived at | In the method, design section |
| Quantitative variables | 11 | Explain how quantitative variables were handled in the analyses. If applicable, describe which groupings were chosen and why | In the method, instruments and statistical analysis section |
| Statistical methods | 12 | (a) Describe all statistical methods, including those used to control for confounding | In the method, statistical analysis section |
|  |  | (b) Describe any methods used to examine subgroups and interactions |  |
|  |  | (c) Explain how missing data were addressed |  |
|  |  | (d) Cross-sectional study—If applicable, describe analytical methods taking account of sampling strategy |  |
|  |  | (e) Describe any sensitivity analyses |  |
| **Results** |  |  |  |
| Participants | 13 | (a) Report numbers of individuals at each stage of study—eg numbers potentially eligible, examined for eligibility, confirmed eligible, included in the study, completing follow-up, and analysed | In the results, the section on the characteristics of the participants |
|  |  | (b) Give reasons for non-participation at each stage |  |
|  |  | (c) Consider use of a flow diagram |  |
| Descriptive data | 14 | (a) Give characteristics of study participants (eg demographic, clinical, social) and information on exposures and potential confounders | In the results, the section on the characteristics of the participants |
|  |  | (b) Indicate number of participants with missing data for each variable of interest |  |
| Outcome data | 15 | Cross-sectional study—Report numbers of outcome events or summary measures |  |
| Main results | 16 | (a) Give unadjusted estimates and, if applicable, confounder-adjusted estimates and their precision (eg, 95% confidence interval). Make clear which confounders were adjusted for and why they were included | In the results, in the tables |
|  |  | (b) Report category boundaries when continuous variables were categorized |  |
|  |  | (c) If relevant, consider translating estimates of relative risk into absolute risk for a meaningful time period |  |
| Other analyses | 17 | Report other analyses done—eg analyses of subgroups and interactions, and sensitivity analyses |  |
| **Discussion** |  |  |  |
| Key results | 18 | Summarise key results with reference to study objectives | In the conclusion, from the third to the sixth paragraph |
| Limitations | 19 | Discuss limitations of the study, taking into account sources of potential bias or imprecision. Discuss both direction and magnitude of any potential bias | In the conclusion, the tenth paragraph |
| Interpretation | 20 | Give a cautious overall interpretation of results considering objectives, limitations, multiplicity of analyses, results from similar studies, and other relevant evidence | In the conclusion, paragraphs nine and eleven |
| Generalisability | 21 | Discuss the generalisability (external validity) of the study results |  |
| **Other information** |  |  |  |
| Funding | 22 | Discuss the generalisability (external validity) of the study results |  |
| *Give information separately for cases and controls in case-control studies and, if applicable, for exposed and unexposed groups in cohort and cross-sectional studies. | | | |
